# A Giant in Plain Sight: Multimodality Imaging of a Left Ventricular Fibroma

**DOI:** 10.7759/cureus.115105

**Published:** 2026-08-24

**Authors:** Michael Sammaan, Michael Moore, Suhair Shebani

**Affiliations:** 1 Paediatric Cardiology, East Midlands Congenital Heart Center, University Hospitals of Leicester, Leicester, GBR

**Keywords:** cardiac computed tomography, cardiac fibroma, implantable cardioverter-defibrillator, speckle-tracking echocardiography, three-dimensional reconstruction

## Abstract

A 14-year-old survivor of out-of-hospital ventricular fibrillation arrest with a large intramural left ventricular fibroma and a secondary-prevention implantable cardioverter-defibrillator underwent multimodality reassessment after device lead fracture. Left ventricular ejection fraction was mildly reduced, whereas speckle-tracking echocardiography demonstrated near-akinetic deformation within tumour-bearing segments. Cardiac computed tomography and three-dimensional reconstruction showed the left circumflex coronary artery and first obtuse marginal branch coursing directly over the mass, with additional proximity to the lateral mitral apparatus. These findings demonstrated substantial regional mechanical impairment while defining the hazards of complete resection. Following multidisciplinary review, device protection and imaging surveillance were favoured over high-risk surgery. This case illustrates how complementary functional and anatomical imaging can resolve a complex management dilemma in ventricular fibroma.

## Introduction

Cardiac fibromas are rare benign tumours and the second most common primary cardiac tumour of childhood. Despite benign histology, they carry a disproportionate risk of ventricular arrhythmia and sudden cardiac death, particularly when large or located within critical myocardial regions [1,2]. Fibromas most often arise within the left ventricular free wall or interventricular septum and typically present as a solitary, intramural mass. On imaging, they characteristically demonstrate attenuation similar to myocardium with focal calcification on CT and low signal on T2-weighted magnetic resonance imaging with variable delayed enhancement; these features, together with an absence of significant vascularity, help to distinguish fibroma from other paediatric cardiac masses such as rhabdomyoma and malignant tumours. Their arrhythmogenic potential arises at the interface between tumour and viable myocardium, where interdigitating tongues of surviving myocardium create a substrate for re-entry. Complete resection may remove the arrhythmogenic substrate, but its potential benefit must be balanced against irreversible ventricular dysfunction, coronary injury, and damage to the mitral apparatus. Conventional measures of global ventricular function may also underestimate the regional mechanical consequences of an intramural mass. We describe an adolescent with a large left ventricular fibroma in whom speckle-tracking echocardiography and cardiac computed tomography (CT) with three-dimensional reconstruction provided complementary information that directly informed management. To our knowledge, the integrated use of regional deformation imaging together with three-dimensional CT coronary and mitral mapping to arbitrate between surgical resection and continued device-based protection in a large left ventricular fibroma has not previously been reported.

## Case presentation

Clinical history and device presentation

A 14-year-old adolescent (weight 50.7 kg; height 162.5 cm) had survived an out-of-hospital ventricular fibrillation arrest at seven years and three months of age. Transthoracic echocardiography at that time identified a large intramural mass within the left ventricular free wall with features consistent with a cardiac fibroma; on serial imaging over the subsequent seven years, the tumour remained stable in size, without interval change. Following the index event, an implantable cardioverter-defibrillator (ICD) was implanted for secondary prevention, comprising a transvenous superior vena cava lead for sensing and a subcutaneous defibrillation coil. The patient remained clinically stable on long-term beta-blocker therapy, with no symptoms of heart failure and one previous inappropriate ICD shock.

The patient was subsequently admitted after routine device surveillance demonstrated high lead impedance, and chest radiography confirmed lead fracture. Because revision of the ICD system was required, the opportunity was taken to reassess tumour extent, ventricular function, the mitral valve apparatus, and coronary anatomy in case surgical resection was reconsidered. At presentation, the patient was asymptomatic. Electrocardiography showed sinus rhythm with lateral T-wave abnormalities corresponding to the tumour-bearing myocardium. Ambulatory electrocardiographic monitoring demonstrated intermittent sinus bradycardia, rare atrial and ventricular ectopy, and no sustained ventricular arrhythmia.

Echocardiographic assessment

**Figure 1:**
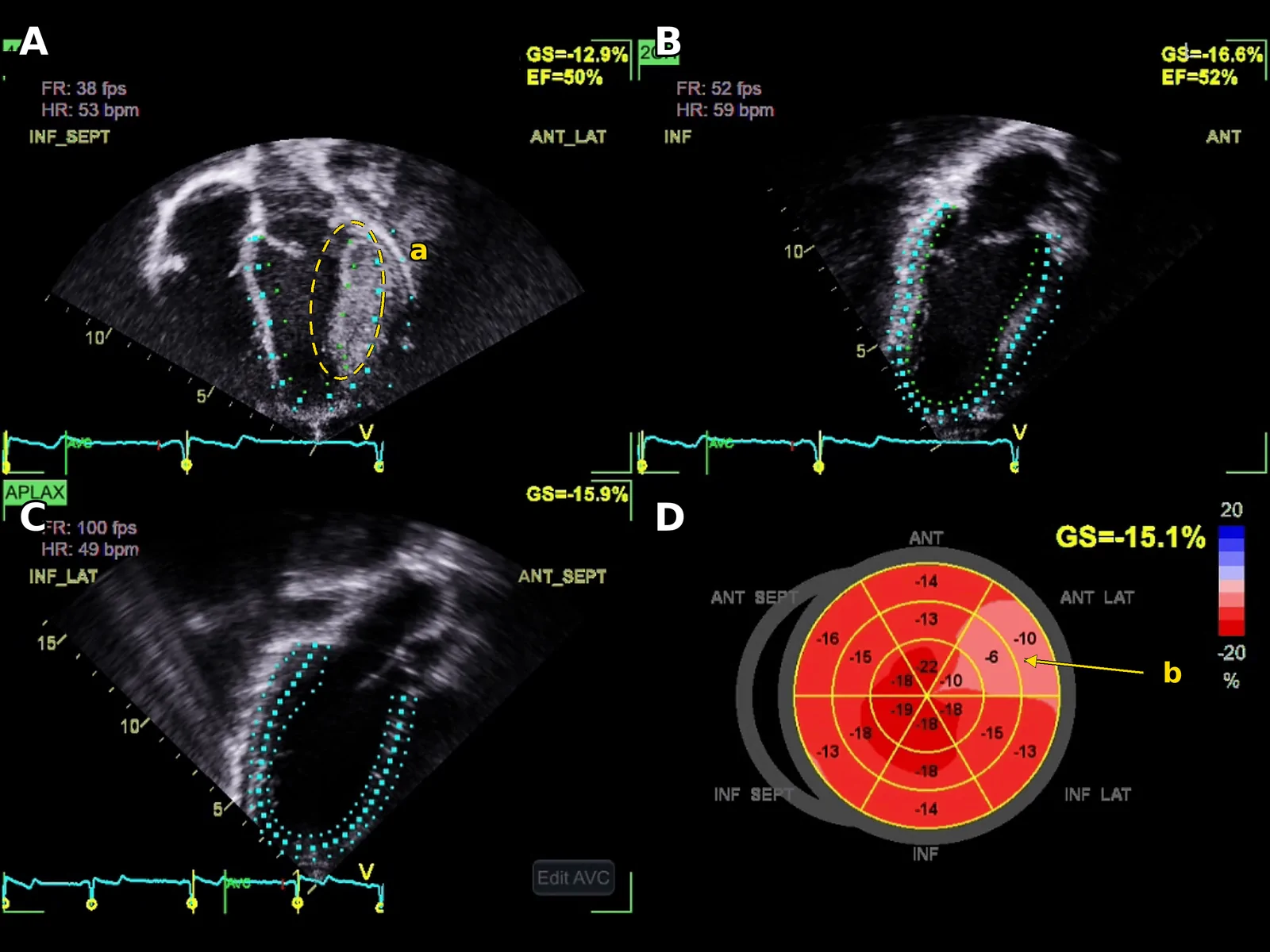
Echocardiographic assessment of regional myocardial deformation Apical four-chamber (A), two-chamber (B), and long-axis (C) speckle-tracking views demonstrate the tumour-bearing left ventricular myocardium. The corresponding 17-segment longitudinal-strain polar map (D) shows abnormal global longitudinal strain of -15.1% and markedly reduced deformation in the anterolateral and inferolateral segments, approaching akinesia at approximately -6% to -11%. Deformation is comparatively preserved in remote inferior and inferoseptal segments at approximately -13% to -22%. Left ventricular ejection fraction was approximately 50%, illustrating discordance between global volumetric function and regional mechanical impairment. The yellow dashed outline (a) delineates the intramural fibroma on the apical four-chamber view; the yellow arrow (b) indicates the tumour-bearing anterolateral segment with markedly reduced longitudinal strain on the polar map. EF, ejection fraction; GS, global strain.

Transthoracic echocardiography demonstrated a large intramural mass involving the anterior, anterolateral, and inferolateral left ventricular walls and extending from the base towards the apex. A thin residual endocardial myocardial rim, approximately 3 mm in thickness, overlaid the mass. There was no left ventricular inflow or outflow tract obstruction. Left ventricular cavity size was normal, right ventricular size and systolic function were preserved, and there was only trivial mitral regurgitation without leaflet distortion. Biplane left ventricular ejection fraction was mildly reduced at approximately 50%. Two-dimensional speckle-tracking analysis showed abnormal global longitudinal strain of -15.1% (apical four-chamber -12.9%, apical two-chamber -16.6%, and apical long-axis -15.9%). Longitudinal deformation was markedly reduced in the tumour-bearing anterolateral and inferolateral segments, approaching akinesia at approximately -6% to -11%, whereas remote inferior and inferoseptal segments showed comparatively preserved deformation of approximately -13% to -22%. These findings demonstrated substantial regional mechanical impairment that was not fully represented by the global ejection fraction (Figure 1). 

Dynamic apical four-chamber speckle-tracking demonstrated reduced longitudinal deformation in the tumour-bearing lateral left ventricular segments, with comparatively preserved deformation in remote myocardium (Video 1).

**Video 1:** Apical four-chamber speckle-tracking echocardiography Dynamic apical four-chamber speckle-tracking echocardiography demonstrates markedly reduced longitudinal deformation in tumour-bearing lateral left ventricular segments, with comparatively preserved deformation in remote myocardium.

Cardiac computed tomography assessment

**Figure 2:**
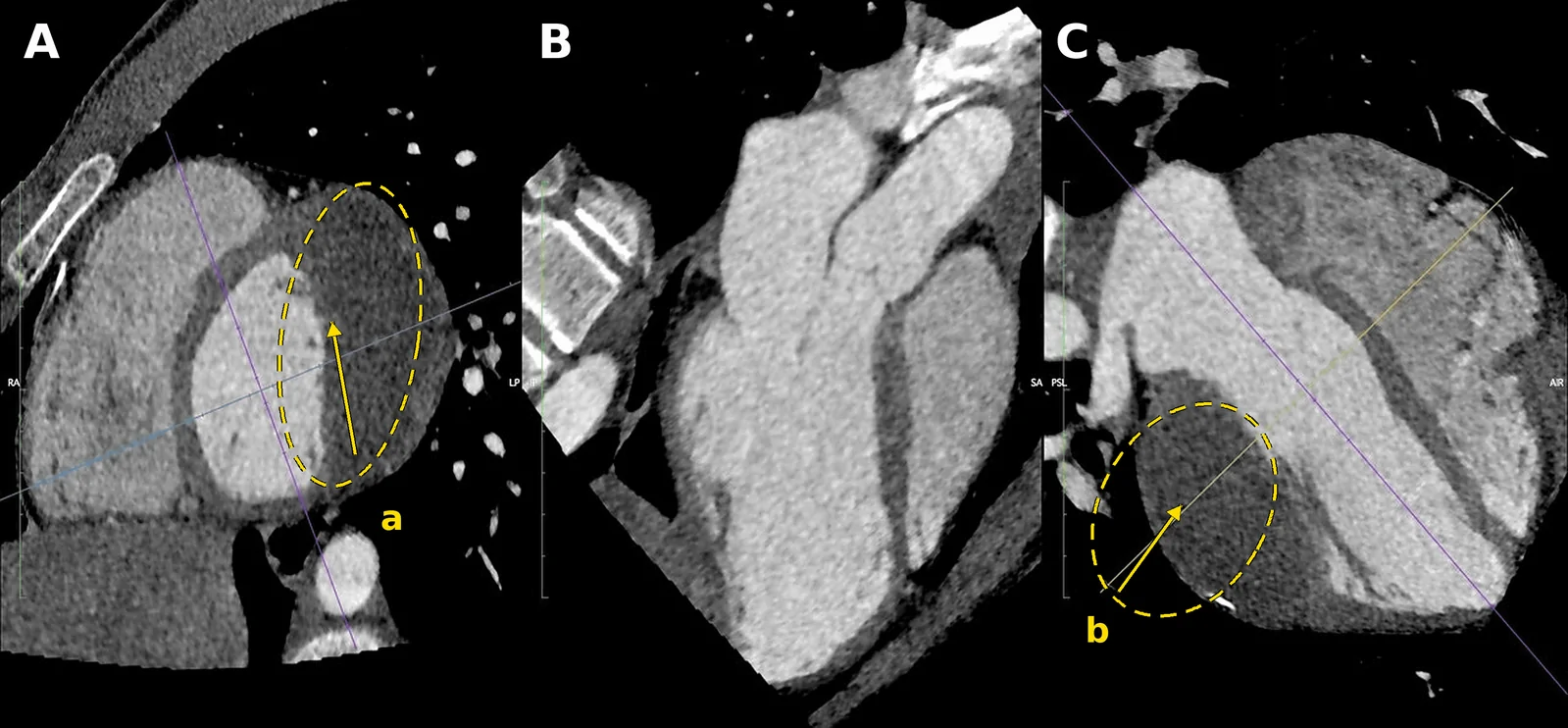
Computed tomography definition of tumour extent Short-axis (A) and orthogonal long-axis multiplanar reformatted images (B and C) demonstrate a 60 × 32 × 53 mm intramural mass arising from the anterior, anterolateral, and inferolateral left ventricular walls. The mass has attenuation similar to adjacent myocardium with focal internal calcification, compresses the left ventricular cavity without causing inflow or outflow tract obstruction, and spares the interventricular septum. A thin residual endocardial myocardial rim overlies the tumour. Yellow dashed outlines and arrows (a, b) delineate the intramural fibroma. CT, computed tomography.

Contrast-enhanced cardiac computed tomography confirmed a large intramural mass arising from the anterior and lateral left ventricular walls and extending from the left atrioventricular groove to within approximately 3 cm of the apex. The mass measured approximately 60 × 32 × 53 mm, had attenuation similar to adjacent myocardium, and contained focal calcification, supporting the imaging diagnosis of cardiac fibroma. It compressed the left ventricular cavity without causing inflow or outflow tract obstruction and spared the interventricular septum (Figure 2).

The coronary origins were normal; however, coronary-focused and volume-rendered reconstructions demonstrated the left circumflex coronary artery and first obtuse marginal branch coursing directly over the epicardial surface of the tumour. The lateral tumour margin was also immediately adjacent to the lateral mitral annulus and subvalvular apparatus. These relationships substantially increased the anticipated risk of complete surgical resection (Video 2).

**Video 2:** Three-dimensional computed tomography reconstruction Rotating three-dimensional volume-rendered cardiac computed tomography reconstruction demonstrates the intramural extent of the left ventricular fibroma and the left circumflex coronary artery with its first obtuse marginal branch coursing directly over the tumour surface. The reconstruction also demonstrates the proximity of the tumour to the lateral mitral annulus and subvalvular apparatus. CT, computed tomography.

Multidisciplinary decision-making and follow-up

The case was reviewed at a joint cardiology and cardiac surgery multidisciplinary meeting. Features favouring resection included the previous ventricular fibrillation arrest, the large tumour burden, and extensive regional mechanical impairment. Conversely, the patient had remained free from sustained ventricular arrhythmia for seven years, had only mildly impaired global left ventricular systolic function, and retained effective secondary-prevention device protection. Most importantly, complete resection was considered to carry substantial risks of left circumflex coronary injury, damage to the lateral mitral apparatus, and postoperative ventricular dysfunction.

An individualised conservative strategy was therefore selected. An automated external defibrillator was provided as interim protection while the fractured system was addressed, followed by revision to a new secondary-prevention ICD system. Over five months of follow-up since device revision, the patient has remained well, with no arrhythmic events or appropriate device therapies, satisfactory device function, and no interval change in tumour size on surveillance. Serial imaging and rhythm surveillance are ongoing.

## Discussion

Cardiac fibromas are an important cause of malignant ventricular arrhythmia in children. Paediatric series have linked adverse outcomes to tumour type, size, and location, and a survivor of ventricular fibrillation with a mass exceeding 50 mm represents a recognised high-risk phenotype [1,2]. The arrhythmogenic substrate is anatomical: interdigitating tongues of viable myocardium at the tumour-myocardium interface create regions of slow conduction and block that can support re-entry [3].

This case demonstrates the limitation of relying on ejection fraction in focal myocardial disease. Ejection fraction is a global volumetric measure and may remain only mildly abnormal when uninvolved myocardium compensates for severely impaired tumour-bearing segments. Speckle-tracking echocardiography showed near-akinetic deformation across the anterolateral and inferolateral walls, providing a functional correlate to the known arrhythmogenic substrate [4,5]. This finding should not be interpreted as a validated independent predictor of ventricular arrhythmia in fibroma; rather, it characterised the regional mechanical consequences of the tumour that were concealed by the global ejection fraction.

Cardiac CT with three-dimensional reconstruction provided the decisive anatomical information. Surgical series support resection when feasible, but extensive transmural involvement and a large tumour volume relative to ventricular size are associated with postoperative ventricular dysfunction [6-8]. In this patient, the left circumflex artery and first obtuse marginal branch ran directly over the mass, while the lateral tumour margin approached the mitral annulus and subvalvular apparatus. These relationships made complete resection potentially hazardous and could not be adequately conveyed by echocardiography alone.

Although multimodality imaging of cardiac fibroma has been described previously, prior reports have largely emphasised anatomical characterisation. The present case illustrates how regional deformation imaging and three-dimensional CT can be combined to weigh functional burden against operative hazard within a single management decision, extending the existing multimodality imaging literature.

Management of ventricular fibroma should therefore be individualised rather than determined by tumour size alone. In this case, strain imaging defined the regional functional burden, whereas CT and three-dimensional reconstruction defined the operative hazard. When integrated with the prolonged absence of recurrent sustained arrhythmia and continued ICD protection, these findings supported surveillance and device-based protection rather than immediate high-risk resection [9].

This report has limitations. The observations derive from a single patient, and the prognostic value of regional strain in cardiac fibroma remains unvalidated; the strain findings are best regarded as characterising regional mechanical impairment rather than as an established predictor of arrhythmic risk or a determinant of surgical candidacy. The follow-up interval since device revision is also relatively short. These hypotheses require validation in larger prospective cohorts.

## Conclusions

In ventricular fibroma, ejection fraction may underestimate severe regional mechanical impairment. Speckle-tracking echocardiography can characterise the functional burden of tumour-bearing myocardium, whereas cardiac CT and three-dimensional reconstruction can define coronary and mitral relationships that determine operative risk. Integrating these modalities may support individualised decisions regarding whether to pursue resection or continue device-based protection with surveillance.
